# Factors that influence the levels of cerebrospinal fluid biomarkers in memory clinic patients

**DOI:** 10.1186/s12877-017-0611-4

**Published:** 2017-09-11

**Authors:** Anne-Brita Knapskog, Rannveig Sakshaug Eldholm, Anne Braekhus, Knut Engedal, Ingvild Saltvedt

**Affiliations:** 1 Department of Geriatric Medicine, The memory clinic, Oslo University Hospital, Ullevaal, Postboks 4956, Nydalen, 0424 Oslo, Norway; 2 Department of Neuromedicine and Movement Science, Norwegian University of Science and Technology (NTNU), Trondheim, Norway; 3 Norwegian National Advisory Unit on Ageing and Health, Vestfold Hospital Trust, Postboks 2136, 3103 Tønsberg, Norway; 4 Department of Geriatrics, St. Olav Hospital, University Hospital of Trondheim, Trondheim, Norway

**Keywords:** Cerebrospinal fluid biomarkers, Alzheimer’s Dementia, Mild cognitive impairment, Memory clinic

## Abstract

**Background:**

The cerebrospinal fluid (CSF) biomarkers amyloid β (Aβ), phospho tau (P-tau) and total tau (T-tau) are used increasingly to support a clinical diagnosis of Alzheimer’s disease. The diagnostic power of these biomarkers has been reported to vary among different studies’ results. The results are poorer when heterogeneous groups of patients have been included compared to studies where patients with Alzheimer’s dementia (AD) and healthy controls have been studied. The aim of this study was to examine if age, APOE genotype and sex were associated with the levels of CSF biomarkers among patients referred to a memory clinic.

**Methods:**

We included 257 patients from two memory clinics who had been assessed for dementia, including lumbar puncture.

**Results:**

The mean age of the patients was 68.1 (SD: 8.0) years; 50.2% were women and 66.5% were APOE ε4 positive. Of these patients, 80.5% were diagnosed with AD or amnestic MCI. Both APOE ε4 and increasing age were associated with decreasing levels of Aβ, but not the levels of the tau proteins. In multiple regression analyses, disease stage, defined as a MMSE ≥25 or <25, influenced factors associated with the CSF biomarkers. Among those with MMSE score ≥ 25, age, APOE ε4 genotype, and MMSE score, in addition to a diagnosis of AD, were associated with Aβ level, with an explained variance of 0.43. When using P-tau or T-tau as a dependent variable, the presence of one or two APOE ε4 alleles, and MMSE score influenced the results, in addition to the diagnosis of AD. The explained variance was lower for P-tau (0.26) and for T-tau (0.32). Among those with MMSE <25, these variables explained very little of the variance. There were no gender differences.

**Conclusions:**

We found that factors in addition to a diagnosis of AD, were associated with the levels of CSF biomarkers. Among those with MMSE ≥25, lower levels of Aβ were associated with several factors including increasing age. This is not reflected in clinical practice, where age-specific cutoffs exist only for T-tau. In this study, age was not associated with the levels of tau proteins.

## Background

Alzheimer’s dementia (AD) is the most common cause of dementia, with an increasing incidence as the world’s population is getting older [1, 2]. Approximately 47 million people suffer from dementia worldwide, and there are around 10 million new cases every year. AD contributes to 60 to 70% of these cases [3]. Several pathological changes are seen in the brains of patients with AD, but none of these changes have, as yet, proven to be specific markers of the disease. AD is still a clinical diagnosis according to various criteria, including the NINCDS-ADRDA criteria [4]. Complemental examinations such as magnetic resonance imaging brain scans (MRI) or positron emission tomography (PET) and cerebrospinal fluid (CSF) biomarkers are being performed increasingly for verification of the diagnosis.

According to the amyloid cascade hypothesis, the disease starts with the deposition of extracellular amyloid plaques, followed by phosphorylation of the tau protein within the neurons, which results in the formation of neurofibrillary tangles and cell loss [5, 6]. Additionally, vascular changes, inflammation, and oxidative stress reactions are seen [7]. The amount of CSF amyloid β (Aβ) is considered to inversely reflect the amyloid burden in the brain. However, a decreased level of Aβ in the CSF is seen not only in AD, but also with increasing age and in patients with dementia with Lewy Bodies (DLB) and vascular dementia (VaD) [8].

The increasing deposition of amyloid in the brain with advancing age is observed not only in people with cognitive impairment but also in individuals with normal cognition [9]. Among people in their 50s and 60s, hardly any amyloidosis is found, while by 80 to 90 years of age, nearly half of all healthy people have both amyloidosis and neurodegenerative changes (such as hippocampal atrophy) in the brain, as verified by MRI and PET [10].

Furthermore, increased levels of total tau (T-tau) and phosphorylated tau (P-tau) in the neurons in the brain result in increased levels of T-tau and P-tau in the CSF of typical patients diagnosed with AD [11]. It has been suggested that both the changes in tau and Aβ in the CSF may precede clinical symptoms by decades [12, 13].

Apolipoprotein E (APOE) is an important transporter of cholesterol in the brain. It also plays a significant role in amyloid metabolism and in the clearance of amyloid [14, 15]. There are three isoforms of APOE (ε2, ε3, and ε4), and APOE ε4 has the poorest capacity for transporting cholesterol and clearing Aβ in the brain [16]. In people who are APOE ε4 positive, more amyloidosis is found [9, 10], meaning there is an additive effect of age and APOE ε4 [17]. This is found in postmortem studies of cognitively intact older people as well [13]. Several studies have shown lower levels of CSF Aβ in APOE ε4-positive people both with and without dementia, and there seems to be a genetic dose-dependent correlation with the lowest levels in the homozygote group [18, 19].

In addition, APOE ε4 has Aβ independent neurotoxic effects including formation of neurotoxic fragments caused by deviant proteolysis of APOE ε4 and, thereby, increased phosphorylation of tau and impaired mitochondrial function [15].

APOE ε4 is the strongest known genetic risk factor for sporadic AD [20, 21]. Worldwide, approximately 15% of all people have one or two alleles of APOE ε4, but the prevalence is found to be higher in Northern Europe [22]. The majority of APOE ε4 carriers are heterozygote (2/4 or 3/4) carriers, and only a small percentage are homozygote (4/4) carriers. People who are heterozygote carriers for APOE ε4 have a 20% to 30% lifetime risk of developing AD, whereas homozygote carriers have a lifetime risk of approximately 50% [23]. This risk of developing AD associated with APOE ε4 is higher for women than for men [23].

Over the last 20 years, there has been a considerable amount of research focused on the diagnostic power of CSF biomarkers. Comparing patients with AD to healthy controls, CSF biomarkers are shown to differentiate healthy persons from those who have the disease very accurately, with reported sensitivity (SS) of 85–94%, and specificity (SP) of 83–100% [24]. Comparing patients with AD to patients with other dementia disorders, the results are less convincing, with lower SS and SP [25]. When keeping SS at a level of 85%, an SP between 39% and 90% is reported in various studies [26]. Thus, the aim of the present study is to examine the influence of factors other than a diagnosis of AD on the levels of CSF biomarkers among a heterogeneous sample of patients referred to a memory clinic.

## Methods

### Aim

Based on the literature, we suggest that age, sex, and APOE genotype may influence the levels of CSF biomarkers of AD in a significant way, especially for the level of Aβ in the CSF.

### Design

The study is a cross-sectional study performed among patients referred to two university memory clinics for diagnostic assessment of dementia in Norway.

### Participants

We included data from the first visits of 257 patients referred from their primary care doctors to the memory clinics at Oslo University Hospital (OUH), Ullevaal (156 patients), and St. Olav University Hospital (97 patients), Trondheim, in the period from October 2009 to January 2015. The only inclusion criteria for participation were that the patient, in addition to the clinical examination, had undergone lumbar puncture for measurement of Aβ and tau proteins, and that the APOE genotype was known. There were no exclusion criteria.

### Examination and procedures for diagnoses

All patients were examined in a standardized and comprehensive manner according to a research protocol [27]. Clinical information was obtained from the patients, their caregivers, their general practitioners, and hospital records. The patients were examined by trained physicians and underwent a neuropsychological examination (including, among others, the Mini-Mental State Examination (MMSE), the Consortium to Establish a Registry of Alzheimer’s Disease [CERAD] 10-item word list, the Clock Drawing Test [CDT], and the Trail Making Tests A and B [TMT A and B]). In addition, physical examinations including blood and spinal fluid samples and magnetic resonance imaging brain scans (MRI) were performed. In some cases, single-photon emission computer tomography (SPECT), DaTscan (Ioflupane I 123 injection), or ^18^F-2-fluoro-2-deoxy-D-glucose positron emission tomography (FDG-PET) was performed when indicated in order to reach a specific diagnosis.

### Spinal fluid biomarkers

The two hospitals performed lumbar punctures and collection of spinal fluid according to an identical procedure. The lumbar punctures were carried out between 9 a.m. and 11 a.m. Spinal fluid was collected in cryotubes and, within thirty minutes of collection, centrifuged for ten minutes at 2000 G. All the samples were analyzed at the same laboratory at Akershus University Hospital (AHUS). Samples were either sent to the laboratory on the same day or frozen at −20 degrees Celsius and later sent in a frozen state. All samples were analyzed with the Innotest kit (Innogenetics, Ghent, Belgium) for all three biomarkers. The laboratory follows its own internal quality control program by using control samples in every batch of samples analyzed. It is also part of the Alzheimer’s Association QC program for CSF biomarkers [28]. The Clinical Neurochemistry Laboratory in Gothenburg, Sweden, is in charge of the program in collaboration with the Alzheimer’s Association. The laboratory at AHUS receives samples from this program for analysis three times a year. The cutoffs for the three biomarkers recommended from the laboratory were applied: amyloid-β (Aβ_42_): > 550, phospho tau (P-tau): < 80, and total tau (T-tau): < 300 for patients under 50 years, < 450 for patients ages 50 to 69, and <500 for those 70 years and older. Age-specific cut offs exist for total tau only.

### APOE genotyping

APOE genotyping was performed using the Illumina Infinium OmniExpress v1.1 chip at deCODE Genetics, Reykjavik, Iceland for all included patients.

### Diagnostic criteria and diagnoses

All patients included in this study had a cognitive impairment. For the diagnosis of mild cognitive impairment (MCI), the Winblad criteria were used [29]. The MCI group was further divided into two groups, amnestic MCI and non-amnestic MCI. Amnestic MCI was defined as patients with MCI with memory complaints as expressed by the patient and/or caregiver and a score equivalent to or below −1.5 SD on the CERAD 10-item delayed recall test, with no other obvious aetiology for cognitive impairment, including MRI findings.

The ICD-10 criteria for research were used to diagnose the patients with dementia. Diagnoses of Alzheimer’s dementia (AD), vascular dementia (VaD), and dementia related to Parkinson’s disease (PDD) were made according to the ICD-10 criteria for research as well [30]. The Manchester–Lund criteria [31] were applied for frontotemporal dementia (FTD) and for dementia with Lewy Bodies (DLB); the consensus criteria according to McKeith et al. [32] were used.

### Statistics

The statistics were carried out using IBM SPSS, version 22. Descriptive analyses of the whole sample and the subgroups MCI, AD, and other dementias were performed (mean, SD, %). As the main aim of the study was to examine factors that could influence the ability of CSF markers to differentiate AD from other disorders, in the further analyses we dichotomized the patients into two groups according to their clinical diagnoses, 207 patients with AD or amnestic MCI and 50 patients with other diagnoses (other MCI, and other forms of dementia). In the multiple linear regression analyses, unadjusted and adjusted analyses were carried out with the three spinal fluid biomarkers as dependent variables. As independent variables, we included age, sex, diagnoses, APOE genotype, and cognitive tests (MMSE and TMT B). Before conducting the adjusted regression analyses, we checked for intercorrelations between the independent variables using the Spearman correlation coefficient. Variables that intercorrelated with a value higher than 0.6 were not included in the analyses. Thus, the CDT, CERAD’s 10-item word list and TMT A had to be excluded from further analyses. The correlation between T-tau and P-tau was high (Spearman’s rho 0.91) but low between Aβ and T-tau or P-tau (Spearman’s rho −0.33 and −0.3, respectively). We also checked for interaction between disease stage (measured by MMSE) and the other predictors included in the regression analyses, and found that the association of the predictors on the biomarkers was dependent on disease stage. In the further analyses we decided to divide the patients into two groups, patients with a MMSE score ≥ 25 and patients with a MMSE score < 25. Multiple regression analyses were performed using both standard and stepwise (enter and backward) methods. The results were the same, and the results using the backward method will be presented. Independent variables with *P* values <0.2 were included in the adjusted analyses.

## Results

### Characteristics of the patients

Of the 257 patients included, 129 (50.2%) were women. The mean age for the whole group was 68.1 years (SD: 8.0; range 43–84 years) with no difference in age between women and men (68.0 [SD: 7.1] vs. 68.2 [SD: 8.6] years respectively, *p* = 0.86). The mean years of education were 12.4 (SD: 3.6); and the mean MMSE score was 23.9 (SD: 4.4). See Table 1 for further details regarding the characteristics of the subgroups of the patients.

**Table 1:** Characteristics of the patients

	MCI patients (*N* = 67)	AD patients (*N* = 165)	Other dementias (*N* = 25)	P
Patient characteristics				
Age [mean (SD)]	66.7 (9.3)	68.7 (7.1)	67.8 (9.3)	0.22^a^
Women [n (%)]	34 (50.7)	87 (52.7)	8 (32.0)	0.15^b^
Education [mean (SD)]	12.8 (4.0)	12.2 (3.4)	12.3 (3.9)	0.54^a^
ApoE ε4 positive [n (%)]	40 (59.7)	118 (71.5)	13 (52.0)	0.06^b^
Cognition				
MMSE [mean (SD)]	26.9 (3.0)	22.7 (4.4)	24.2 (3.8)	<0.001^c^
CDT accepted [n (%)]	54 (83.1)	76 (47.5)	15 (60.0)	<0.001^b^
TMT A ≥ −2 SD [n (%)]	55 (83.3)	89 (58.0)	10 (41.7)	<0.001^b^
TMT B ≥ −2 SD [n (%)]	45 (69.2)	57 (41.0)	7 (31.8)	<0.001^b^
CSF biomarkers				
Aβ [mean (SD)]	799.1 (315.6)	583.3 (210.5)	735.9 (281.7)	<0.001^c^
T-Tau [mean (SD)]	464.7 (260.8)	717.0 (357.9)	305.1 (165.4)	<0.001^c^
P-tau [mean (SD)]	63.6 (24.4)	83.6 (35.0)	47.3 (16.7)	<0.001^c^

*MCI* mild cognitive impairment, *AD* Alzheimer’s Disease, *MMSE* Mini Mental State Examination, *CDT* the Clock Drawing Test, *TMT* Trail Making Test A and B, *Aβ* Amyloid β, *T-tau* total tau, *P-tau* phosho tau

% = valid percent without missing

^a^one-way ANOVA, ^b^Chi square, ^c^Kruskal-Wallis test

The patients recruited from St. Olav University Hospital differed from the patients at OUH in regard to age, education, and cognitive impairment. They were older (mean 72.8 years [SD: 4.6] vs. 65.2 years [SD: 8.2] *p* < 0.001); they had a lower level of education (mean 10.4 years [SD: 2.9] vs. 13.6 years [SD: 3.5] *p* < 0.001); and they had significantly lower scores on the MMSE (median 23.0 vs. 26.0, *p* = 0.001) and on the delayed-recall test of the CERAD’s 10-item word list (mean 1.7 words [SD: 1.9] vs. 2.3 words, [SD 2.2] *p* = 0.05).

There was a significant difference between the patient group with a MMSE score < 25 compared with the group with a MMSE score ≥ 25. In the group with MMSE scores <25, mean age was higher (69.4 [SD: 7.2] vs.67.1 years [8.5] *p* = 0.02); there were more women (61.3% vs. 41.0%, *p* = 0.001); the diagnosis of AD/aMCI was more frequent (89.9% vs. 73.1%, *p* = 0.001). In addition; the CSF biomarkers were more pathological (Aβ mean 571.5 [SD: 200.9] vs. 729.8 [SD: 296.2] *p* < 0.001, P-tau mean 80.3 [SD: 34.9] vs. 70.7 [SD: 31.8] *p* = 0.03, and T-tau mean 693.6 [SD: 358.7] vs. 545.5 [SD: 334.9] p < 0.001).

### Diagnoses

Of the whole group of 257 patients, 67 (26.1%) patients were diagnosed with MCI; 165 (64.2%) had AD (including 27 [10.5%] patients with mixed AD/VaD) with typical AD symptomatology. Of the remaining 25 (9.7%) patients, 1 (0.4%) had VaD; 3 (1.2%) had FTD; 14 (5.5%) were diagnosed with PDD/DLB; and 7 (2.8%) had other dementias. Of the 67 patients with MCI, 42 (62.7%) were classified as patients having amnestic MCI. The remaining 25 (37.3%) patients met the criteria for MCI due to cerebrovascular disease or other physical disorders, psychiatric disorders such as depression, or drug and/or alcohol abuse.

### Associations between the levels of Aβ, diagnosis, and APOE genotype in different age groups

Of the whole study population, 66.5% were APOE ε4 carriers; 48.2% were heterozygote; and 18.3% were homozygote. The prevalence of none, one, or two APOE ε4 alleles differed in the three different age groups. In the youngest group (below 65 years of age), approximately 58% were heterozygote or homozygote, whereas in the groups older than 65 years, approximately two-thirds were APOE ε4 carriers. There was no gender difference in the prevalence of the APOE ε4 allele (*p* value 0.29) or between patients with AD and patients with amnestic MCI (71.5% vs. 66.7% positive, *p* = 0.54). Among patients with AD and amnestic MCI, the level of Aβ was significant lower in APOE ε4 carriers in the two oldest age groups. There was an age-dependent decrease in the CSF Aβ in non-carriers and in patients with one ε4 allele, while this was not statistically significant in homozygote patients. See Table 2 for further details. A similar age-dependent increase in the CSF P-tau or T-tau was not shown in any of the groups. However, in the group 65–74 years the levels of P-tau and T-tau increased with the highest levels in homozygote carriers (*p* values 0.009 and 0.004, respectively).

**Table 2:** Associations between the levels of Aβ, diagnosis, and ApoE genotype in different age groups

	Alzheimer’s Dementia/amnestic mild cognitive impairment						
	Age < 65 years (*N* = 48)		Age 65–74 years (*N* = 110)		Age 75 years and older (*N* = 49)		
ApoE ε4	N (%)	Aβ [mean (SD)]	N (%)	Aβ [mean (SD)]	N (%)	Aβ [mean (SD)]	*p*
None	20 (41.7)	774.1 (366.0)	23 (20.9)	687.4 (285.7)	18 (36.7)	637.4 (264.4)	0.02^a^
Heterozygote	17 (35.4)	659.1 (238.3)	61 (55.5)	596.9 (173.8)	24 (49.0)	528.8 (123.8)	0.01^a^
Homozygote	11 (22.9)	547.2 (113.0)	26 (23.6)	498.9 (99.9)	7 (14.3)	433.9 (116.1)	0.37^a^
P		p = 0.21^a^		*p* = 0.01^a^		*p* = 0.07a	

*Aβ* amyloid-β

^a^Kruskal-Wallis test

### Multiple regression

For all three CSF biomarkers, multiple regression analyses gave different results for patients with MMSE <25 as compared to patients with MMSE ≥25. Among patients with a MMSE score ≥ 25, most of the variance for Aβ could be explained by other variables than the diagnosis of AD. For P-tau and T-tau, more of the variance was explained by the diagnosis of AD; see Tables 3, 4 and 5.

**Table 3:** Multiple linear regression - Amyloid β

Variables	Unadjusted standardized beta	P	R square	Adjusted standardzied beta	P
Patients with MMSE sum score ≥ 25, *N* = 134					
Diagnoses (AD/aMCI = 2, others = 1)	−0.33	<0.001	0.1	−0.18	0.02
Age	−0.41	<0.001	0.16	−0.19	0.02
Sex (1 = women, 2 = men)	0.25	0.007	0.05	0.13	0.07
ApoE ε4 genotype (neg = 1, pos = 2)	−0.53	<0.001	0.27	−0.41	<0.001
MMSE	0.34	<0.001	0.1	0.21	0.004
R square				0.43	
Patients with MMSE sum score < 25, *N* = 119					
Age	−0.14	0.13	0.01		
Sex (1 = women, 2 = men)	−0.09	0.34	−0.001		
ApoE ε4 genotype (neg = 1, pos = 2)	−0.12	0.19	0.006		
MMSE	0.14	0.13	0.01		
R square				0.0	

*AD/aMCI* Alzheimer’s dementia/amnestic mild cognitive impairment

MMSE missing in 4 patients

**Table 4:** Multiple linear regression - phospho tau

Variables	Unadjusted standardized beta	P	R square	Adjusted standardized beta	P
Patients with MMSE sum score ≥ 25, *N* = 134					
Diagnoses (AD/aMCI = 2, others = 1)	0.42	<0.001	0.17	0.36	<0.001
Age	0.11	0.2	0.005		
Sex (1 = women, 2 = men)	−0.18	0.04	0.03		
ApoE ε4 genotype (neg = 1, pos = 2)	0.23	0.007	0.05	0.17	0.03
Amyloid β	−0.35	<0.001	0.11		
MMSE	−0.33	<0.001	0.1	−0.25	0.002
R square, adjusted				0.26	
Patients with MMSE sum score < 25, *N* = 119					
Age	0.12	0.18	0.007		
Sex (1 = women, 2 = men)	0.006	0.95	−0.009		
ApoE ε4 genotype (neg = 1, pos = 2)	0.17	0.07	0.02		
Amyloid β	−024	0.01	0.05	−0.24	0.01
MMSE	−017	0.06	0.02		
R square, adjusted				0.05	

*AD/aMCI* Alzheimer’s dementia/amnestic mild cognitive impairment

MMSE missing in 4 patients

**Table 5:** Multiple linear regression - total tau

Variables	Unadjusted standardized beta	P	R square	Adjusted standardized beta	P
Patients with MMSE sum score ≥ 25, *N* = 134					
Diagnoses (AD/aMCI = 2, others = 1)	0.44	<0.001	0.19	0.36	<0.001
Age	0.12	0.16	0.007		
Sex (1 = women, 2 = men)	−0.17	0.05	0.02		
ApoE ε4 genotype (neg = 1, pos = 2)	0.26	0.002	0.06	0.2	0.008
Amyloid β	−0.36	<0.001	0.12		
MMSE	−0.39	<0.001	0.15	−0.31	0.001
R square				0.32	
Patients with MMSE sum score < 25, *N* = 119					
Age	0.07	0.46	−0.004		
Sex (1 = women, 2 = men)	−0.02	0.87	−0.008		
ApoE ε4 genotype (neg = 1, pos = 2)	0.18	0.05	0.03		
Amyloid β	−0.25	0.005	0.06	−0.24	0.01
MMSE	−0.16	0.09	0.02		
R square				0.07	

*AD/aMCI* Alzheimer’s dementia/amnestic mild cognitive impairment

MMSE missing in 4 patients

In the unadjusted linear regression analyses using Aβ as the dependent variable, all the included variables (diagnosis, age, sex, APOE genotype, and MMSE) were significantly associated with Aβ among patients with a MMSE score ≥ 25. In the adjusted analyses, all these variables, except sex, remained associated. The diagnosis of AD, age, the presence of one or two APOE ε4 alleles, and the MMSE score were associated with Aβ with an explained variance of 0.43.

When using P-tau or T-tau as a dependent variable, the diagnosis of AD, the presence of one or two APOE ε4 alleles, and the MMSE score were significant in the adjusted analyses. The explained variances for P-tau and T-tau were lower than for Aβ (0.26 and 0.32 respectively). See Tables 3, 4 and 5.

In patients with a MMSE score < 25, approximately 90% of the patients had a diagnosis of AD/aMCI, thus diagnoses had to be excluded from the analyses. In the adjusted linear regression analyses using Aβ as the dependent variable, none of the variables in the analyses were associated. Using P-tau or T-tau as the dependent variable, the level of Aβ was the only significant variable. The explained variance was 0.05 for P-tau and 0.07 for T-tau.

## Discussion

In the present descriptive study of memory clinic patients, we studied the association of the CSF biomarkers with other factors than a diagnosis of AD. We found that, in addition to a diagnosis of AD, the age of the patients, the presence of one or two APOE ε4 alleles, and the results on the MMSE were associated with the level of Aβ in the CSF. As for P-tau and T-tau, a diagnosis of AD, the presence of one or two APOE ε4 alleles, and the MMSE score were associated with the levels of both biomarkers. There were no gender differences. These associations were however, only significant among those with less advanced disease (MMSE ≥25). Among the patients with MMSE <25, the predictors included in the analyses explained none or very little of the variance.

In this study, 66.5% of the patients were APOE ε4 carriers, a prevalence that indicated a selected group of patients compared with the general population [22]. The same frequency is found among patients diagnosed with AD in Norway earlier [33]. Analysing the whole group of patients, fewer younger patients were APOE ε4 carriers as compared to the oldest patients of the sample, where two-thirds had at least one APOE ε4 allele. Among patients with AD/aMCI we found an APOE ε4 dose-dependent reduction in Aβ in the CSF among the two oldest age groups (65 years and older), with the lowest level of Aβ among homozygote carriers. There was no association between the APOE genotype and decreased levels of Aβ among the youngest patients (below 65 years of age); see Table 2. The APOE genotype did not play a significant role in the levels of T-tau and P-tau. The association between APOE ε4 status and Aβ, and the lack of associations between APOE ε4 status and the tau proteins, was also reported by Mehrabian et al. in patients with AD. In that study, an association between APOE genotype and CSF Aβ was shown among those with late onset AD (LOAD), but not in early onset AD (EOAD), [20]. The effect of APOE ε4 on Aβ in LOAD was dose dependent, meaning that patients with two alleles had lower levels of Aβ in the CSF than those with one allele [20]. The results of Mehrabian et al. are similar to our results. This may indicate that although APOE ε4 is of importance for the development of LOAD, other factors may be of greater importance among younger patients [14].

The association between APOE ε4 status and Aβ, and the lack of associations between APOE ε4 status and the tau proteins was also reported by Price et al., who conducted a postmortem analysis of cognitively intact old people [13]. His results may be a result of aging, or it may be that many of the people examined postmortem had preclinical AD, which could have been expressed as clinical AD later, as found in a study by Monge-Argiles et al. [34]. In that study where patients with MCI and healthy controls were included, a higher conversion rate from MCI to AD was shown among the oldest patients (> 74 years old) and in APOE ε4 carriers regardless of age [34]. In our study, the association between the APOE genotype and decreased levels of Aβ was found only in the two oldest patient groups of AD/aMCI (65 years and older).

However, the increasing effect of APOE ε4 on Aβ but not on tau pathology with advanced age is found in other studies among people with normal cognition as well [17]. In a previous study by Morris et al., the role of APOE genotype in with advancing age in people without cognitive impairment was explored [17]. They found that both age and APOE ε4 were associated with lower levels of Aβ in older people with normal cognition, and that the deposition of amyloid plaques starts in middle age and increases throughout life (34.2% in the age group 70–79 years, increasing to 50% in the age group 80–89 years, who had reduced levels of Aβ). The effect of age was independent of the presence of APOE ε4 allele [17]. These results are similar to the results in the Price et al. study [13]. In our study there was a significant association between age and Aβ among AD and aMCI patients who had none or one APOE ε4 allele, but not in the patients with two alleles who had lower levels of Aβ in all three age groups; see Table 2. These results are not reflected in the recommended cutoffs of the CSF markers used in clinical practice as there are age specific cutoffs for T-tau, but not for Aβ.

The associations already mentioned were however only valid among the patients with MMSE ≥25 where age influenced the levels of Aβ, but not the levels of T-tau or P-tau in the adjusted regression analysis; see Tables 3, 4 and 5. In addition, a weaker association was found between APOE ε4 and Aβ, P-tau and T-tau among these patients.

In the group of patients with MMSE <25, we could not find any association between the APOE ε4 and Aβ or the tau proteins. In the study by Mehrabian et al., the mean MMSE score was 22.3 (SD: 4.3) which makes it comparable with this group. In their study no differences were found in the CSF levels of Aβ between patients with EOAD vs. LOAD [19], which is in line with our findings.

The women in this study had lower levels of Aβ than the men. However, the women had poorer results on all the cognitive tests, indicating a more severe cognitive impairment in line with the findings of Mehrabian et al. [20]. In the multiple regression analysis, sex was not significant after adjusting for cognition. In a study by Rosen et al., women had lower Aβ and higher of P-tau and T-tau levels as compared to men independent of the degree of cognitive impairment. In that study, the APOE E genotype was not known [35]. As some studies have found that women having a higher APOE ε4 associated risk of developing AD than men, this could have influenced the results of that study [23].

### Strengths

The patients in this study represent the types of patients who are normally referred to our memory clinics and were not selected otherwise. All patients underwent a standardized comprehensive assessment including neuropsychological examination, physical examinations including blood and spinal fluid samples, and supplementary brain imaging.

### Limitations

Although it continues to be emphasized that AD is a clinical diagnosis, both in the revision of the NINCDS-ADRDA and in the IWG-2 revision of the research criteria, clinical diagnoses are not always correct [4, 36]. However, we believe that, in the present study, few patients have been misdiagnosed. Experienced physicians made the diagnoses based upon the comprehensive assessment in order to minimize variations. Another challenge was that in some analyses the groups were too small to achieve significant results.

A further challenge when using CSF biomarkers is interlaboratory and intralaboratory variations, to which Aβ is especially vulnerable [36]. In this study, the same laboratory equipment was used, and the same laboratory analysed all samples to reduce pre-analytical variability.

## Conclusion

In this study conducted among memory clinic patients, we found that in addition to an AD diagnosis, Aβ was associated with age, by the presence of one or two APOE ε4 alleles, and by the MMSE score. As expected, the levels of T-tau and P-tau were both associated with a diagnosis of AD, the MMSE score, but also with by the presence of one or two APOE ε4 alleles. These findings were valid for patients with a MMSE score ≥ 25. Among patients with a MMSE score < 25, these variables explained very little of the variance, meaning that other factors may be of importance. In clinical practice, age specific cutoffs should be considered not only for T-tau, but also for Aβ.

## Abbreviations

AD: Alzheimer’s dementia
AHUS: Akershus University Hospital
aMCI: Amnestic mild cognitive impairment
APOE: Apolipoprotein E
Aβ: Amyloid β
CDT: Clock Drawing Test
CERAD: The Consortium to Establish a Registry of Alzheimer’s Disease
CSF: Cerebrospinal fluid
DaTscan: Dopamine transporter imaging
DLB: Dementia with Lewy Bodies
EOAD: Early onset Alzheimer’s dementia
FDG-PET: 18F-2-fluoro-2-deoxy-D-glucose positron emission tomography
FTD: frontotemporal dementia
ICD-10: International Classification of Diseases
IWG: International Working Group
LOAD: Late onset Alzheimer’s dementia
MCI: Mild cognitive impairment
MMSE: Mini-Mental State Examination
MRI: Magnetic Resonance Imaging
NINCDS-ADRDA: National Institute of Neurological and Communicative Disorders and stroke – Alzheimer’s Disease and Related Disorders Association
OUH: Oslo University Hospital
PDD: Parkinson Disease Dementia
P-tau: Phospho tau
SD: Standard deviation
SP: Specificity
SPECT: Single-photon emission computer tomography
SS: Sensitivity
TMT A and B: Trail Making Tests A and B
T-tau: Total tau
VaD: Vascular dementia
